# Vector Competence of California Mosquitoes for West Nile virus

**DOI:** 10.3201/eid0812.020536

**Published:** 2002-12

**Authors:** Laura B. Goddard, Amy E. Roth, William K. Reisen, Thomas W. Scott

**Affiliations:** * University of California, Davis, California, USA

**Keywords:** West Nile virus, vector competence, mosquito, Culex

## Abstract

To identify the mosquito species competent for West Nile virus (WNV) transmission, we evaluated 10 California species that are known vectors of other arboviruses or major pests: Culex tarsalis, Cx. pipiens pipiens, Cx. p. quinquefasciatus, Cx. stigmatosoma, Cx. erythrothorax, Ochlerotatus dorsalis, Oc. melanimon, Oc. sierrensis, Aedes vexans, and Culiseta inornata. All 10 became infected and were able to transmit WNV at some level. Ochlerotatus, Culiseta, and Aedes were low to moderately efficient vectors. They feed primarily on mammals and could play a secondary role in transmission. Oc. sierrensis, a major pest species, and Cx. p. quinquefasciatus from southern California were the least efficient laboratory vectors. Cx. tarsalis, Cx. stigmatosoma, Cx. erythrothorax, and other populations of Cx. pipiens complex were the most efficient laboratory vectors. Culex species are likely to play the primary role in the enzootic maintenance and transmission of WNV in California.

Three years since its 1999 introduction into North America, West Nile virus (WNV) has spread rapidly from New York to the Rocky Mountains and to the Gulf of Mexico. As of September 2002, over 1,900 human cases of WNV encephalitis have been confirmed with 94 deaths; >6,000 equine cases also occurred during 2002 (1). The imminent spread of this virus culminated in the establishment of WNV surveillance programs in 48 states. Surveillance programs include testing mosquito pools for virus, sentinel chickens for seroconversion, wild birds for virus and seroconversion, and equine and human cases (2).

WNV is a geographically widespread arbovirus in the family Flaviviridae, genus Flavivirus (3). The virus, first isolated from the blood of a woman in the West Nile district of Uganda in 1937 (4), historically has been endemic to Africa, Western Asia, and the Middle East. Recently, WNV has expanded its distribution and caused epidemics in Russia, Romania, France, and Israel (5,6).

WNV is maintained in an enzootic transmission cycle among Culex mosquitoes and wild birds. In Africa and the Middle East, WNV has been most frequently isolated from Cx. univitattus (7,8). In Asia, members of the Cx. vishnui complex have been implicated as the primary vectors (9). Cx. modesuts was identified as a principal vector during a 1960s epidemic in France (3). During the North American outbreak, members of the Cx. pipiens complex were considered the primary epizootic vectors (10). Since the New York outbreak in 2002, WNV has been recovered from 26 North American mosquito species, including Cx. pipiens, Cx. salinarius, Cx. restuans, Ochlerotatus canadensis, Oc. japonicus, Aedes vexans, and Culiseta melanura (11,12). Recent vector competence studies indicate that some North American Culex and Ochlerotatus species are relatively efficient laboratory vectors (13–15).

As WNV expands its range westward across North America, examining the transmission potential of the different mosquito species will help to anticipate patterns of transmission and the relative contribution of different vector species to virus amplification and persistence. The enzootic transmission cycles of WNV, Saint Louis encephalitis virus (SLEV), and Western equine encephalomyelitis virus (WEEV) in North America are conceptually identical, with Culex vectors transmitting virus in passerine avian hosts. In the western United States, SLEV and WEEV share a common mosquito host, Cx. tarsalis, which will presumably also support WNV transmission. Moreover, WNV and SLEV are closely related viruses in the Japanese encephalitis virus (JEV) serocomplex (3), and Cx. tarsalis has been shown to be an efficient vector of both SLEV (16) and JEV (17). Current WNV control strategies are based largely on vector control (18); therefore, identifying which species have the greatest potential for transmission is essential in formulating and focusing a prevention plan (19). We evaluated 10 California vector and pest mosquito species’ for their ability to become infected with and transmit WNV.

## Materials and Methods

### Mosquitoes

We assessed the vector competence for WNV of 10 California mosquito species from 14 different geographic locations (Table 1). Vector competence refers to the intrinsic permissiveness of an arthropod for the infection, replication, and transmission of a virus (20,21). Voucher specimens for each species were deposited at the Bohart Museum of Entomology at the University of California, Davis, California. Cx. tarsalis is the principal enzootic vector of WEEV and SLEV in California (22). Members of the Cx. pipiens complex have been primary vectors of WNV in New York (10) and could potentially play a similar role in California, especially in urban environments. We defined members of the Cx. pipiens complex on the basis of the geographic location of collection and on previously described hybrid zones in California (23). Consequently, we considered members of the complex collected from northern California to be Cx. p. pipiens, and those collected from central and southern California to be Cx. p. quinquefasciatus. Cx. stigmatosoma, an abundant species in California, is naturally infected with WEEV and SLEV (24) and is an efficient laboratory vector of SLEV (16). Cx. erythrothorax, another widespread species, typically inhabits marshlands and is an opportunistic feeder (25). Oc. dorsalis and Oc. melanimon, involved in the transmission of WEEV in small mammals, are laboratory-confirmed vectors of WEEV (26,27). Oc. sierrensis is a major pest in California that frequently bites humans and other mammals and transmits dog heartworm, Dirofilaria immitis (28). We tested Ae. vexans because it feeds readily on mammals (29) and was found to be naturally infected with WNV during the 1999 New York outbreak (11,12). Cs. inornata is a mosquito that is active during the winter; this species could potentially maintain WNV amplification and transmission during winter months when Culex species are inactive (30).

**Table 1:** California mosquito species tested for vector competence for WNV^a^

Species	Source^b^	Generation
*Culex tarsalis*	Yolo Co.	F_1_
	Bakersfield, Kern Co.	F_1_
	Coachella Valley, Riverside Co.	F_1_
*Cx. pipiens quinquefasciatus*	Bakersfield, Kern Co.	F_0_
	Coachella Valley, Riverside Co.	F_1_
	Orange Co.	Wild adults
*Cx. p. pipiens*	Shasta Co.	F_1_
*Cx. stigmatosoma*	Chino, San Bernardino Co	Wild adults
*Cx. erythrothorax*	San Joaquin Marsh, Orange Co.	Wild adults
	Coachella Valley, Riverside Co.	Wild adults
*Ochlerotatus dorsalis*	Morro Bay, San Luis Obispo Co.	F_0_
*Oc. melanimon*	Lost Hills, Kern Co.	Wild adults
*Oc. sierrensis*	Lake Co.	F_0_
*Aedes vexans*	Coachella Valley, Riverside Co.	F_1_
*Culiseta inornata*	Lost Hills, Kern Co.	F_0_

^a^WNV, *West Nile virus*, Co., County; F_0_, adults reared from wild-caught larvae or eggs; F_1,_ progeny from wild-caught adults reared in the laboratory; wild adults, wild-caught adults of unknown age. ^b^All mosquitoes were collected during 2001 except *Cx. p. quinquefasciatus* (Orange Co.), *Cx. stigmatosoma*, *Cx. erythrothorax,* and C*x. inornata*, which were collected during 2002.

### Virus and Virus Assay

We used WNV strain 35211 AAF 9/23/99, which was isolated from a flamingo during the 1999 New York outbreak and passaged twice in Vero (African green monkey kidney) cell cultures. All artificial blood meal, transmission, and mosquito body samples were examined for virus by plaque assay in six-well tissue culture plates (Costar, Corning, NY) containing monolayers of Vero cells. Mosquito bodies were ground individually in 0.5 mL of mosquito diluent (phosphate-buffered saline [PBS], 20% fetal bovine serum [FBS], antibiotics). Plaque assays were conducted by adding 100 µL of each sample to confluent cell monolayers and incubating inoculated cells at 37°C for 1.5 h to allow for virus to attach and enter cells. After incubation, cells were covered with a 2% agarose overlay containing 0.005% neutral red. After 96 h and 120 h of incubation at 37°C, in a 5% CO2 atmosphere, plaques were counted, and virus concentrations were calculated as PFUs per 1.0 mL.

### Mosquito Infection

Mosquitoes were infected orally by feeding on hanging blood droplets (defibrinated rabbit blood [Microbiological Media, San Ramon, CA]) containing 2.5% sugar and 107.1±0.1 or 104.9±0.1 WNV PFUs/1.0 mL of blood. Infectious blood was diluted in bovine albumin-PBS and stored at –80°C until examined by plaque assay to determine the titer. Engorged mosquitoes were held at 28°C, during a 16:8 light:dark photoperiod, and provided a 10% sucrose solution in cotton wicks.

### Experimental Transmission

Mosquitoes were deprived of sucrose for 24 h before transmission attempts. On days 7 and 14 after infection, mosquitoes were immobilized by exposure to triethylamine and their proboscises were inserted into a capillary tube containing a 1:1 FBS and 10% sucrose solution for 10 min (31). Transmission fluid was expelled into 250 µL of mosquito diluent and frozen at –80°C until assayed. Individual mosquito bodies were similarly frozen at –80°C before being thawed, ground, and assayed.

### Statistical Analysis

Infection and transmission rates were compared in day 7 and day 14 data for each dose by the Fisher exact test by using SAS 8.2 (SAS Institute, Inc., Cary, NC). Differences were considered statistically significant at alpha >0.05 and adjusted for multiple comparisons.

## Results

All mosquito species tested were susceptible to infection, and WNV was detected, to some extent, in their salivary secretions. Infection rates were generally higher after 7 days’ incubation than 14 days. Transmission rates were generally highest for females infected with the high dose of 107.1±0.1 PFU/mL and incubated for 14 days (Table 2).

**Table 2:** Infection and transmission rates for California mosquito species orally infected with 10^7.1±0.1^ PFU/mL of *West Nile virus*

Species	Source by county	Day transmission attempted	No. tested	Infection rate^a^	Transmission rate^b^
*Culex tarsalis*	Yolo	7	30	87	60
		14	1	100	100
	Kern	7	15	93	40
		14	35	74	60
	Riverside	7	49	94	10
		14	55	85	62
*Cx. pipiens quinquefasciatus*	Kern	7	50	86	4
		14	50	58	52
	Riverside	7	60	8	0
		7	60	13	2
		14	58	28	19
	Orange	7	45	80	9
		14	50	66	36
*Cx. p. pipiens*	Shasta	7	17	100	0
		14	31	100	71
*Cx. stigmatosoma*	San Bernardino	7	15	67	0
		14	48	77	19
*Cx. erythrothorax*	Orange	7	15	100	33
		14	25	100	64
*Ochlerotatus dorsalis*	Kern	7	30	50	13
		14	29	41	34
*Oc. melanimon*	San Luis Obispo	7	50	46	18
		14	60	48	20
*Oc. sierrensis*	Lake	7	40	5	3
		14	50	14	6
*Aedes vexans*	Riverside	14	22	32	23
*Culiseta inornata*	Kern	14	28	75	21

^a^Percent of mosquito bodies positive for WNV. ^b^Percent of transmission attempts positive for WNV.

Infection rates varied markedly among species but were consistently highest after infection with the high dose of WNV. Infection rates of Culex species and Cs. inornata tested 14 days after imbibing the high virus dose ranged from 58% to 100%, except for Cx. p. quinquefasciatus from the Coachella Valley and Orange County. Oc. dorsalis and Oc. melanimon infection rates ranged from 41% to 48%. Ae. vexans had a moderate infection rate of 32%, whereas Oc. sierrensis and Cx. p. quinquefasciatus from the Coachella Valley had infection rates <15%. The last two infection rates are significantly lower than the day-14 high–dose infection rates for all species tested, except for Cx. tarsalis (Yolo County), Cx. p. quinquefasciatus (Orange County), Oc. dorsalis, and Ae. vexans (p<0.0009). Despite the high susceptibility of Cx. tarsalis (Yolo County), its day-14 infection rates are not statistically significant, which may be attributed to the small sample size.

Culex species, excluding Cx. p. quinquefasciatus from the Coachella Valley and Orange County, were most efficient at transmitting virus after exposure to the high dose and 14-day incubation period. Cx. tarsalis (Yolo County) was the most efficient laboratory vector; 60% of expectorate samples contained virus after only 7 days of incubation. These Cx. tarsalis (Yolo County) transmission results were significantly higher than all other day-7 high–dose transmission rates (p<0.001), except for Cx. tarsalis (Bakersfield) and Cx. erythrothorax (Coachella Valley). Only one Cx. tarsalis (Yolo County) was tested on day 14 because of excessive mortality beginning on day 10. After 14 days of incubation, >60% of the Cx. tarsalis from all three regions in California, Cx. stigmatosoma and Cx. erythrothorax (Coachella Valley) transmitted virus. Cx. p. quinquefasciatus (Bakersfield) followed closely with a 52% transmission rate. Cx. p. quinquefaciatus (Orange County), Cx. p. pipiens, Cx. erythrothorax (Orange County), Oc. dorsalis, Oc. melanimon, Ae. vexans, and Cs. inornata had moderate transmission rates ranging from 19% to 36%. Oc. sierrensis and Cx. p. quinquefasciatus from the Coachella Valley were poor vectors, transmitting virus at rates <6%. Transmission rates for Cx. p. quinquefasciatus (Coachella Valley) were significantly lower than those of Cx. tarsalis (Coachella Valley, Bakersfield), Cx. p. quinquefasciatus (Bakersfield), Cx. p. pipiens, Cx. stigmatosoma, Cx. erythrothorax (Coachella Valley), and Oc. dorsalis (p<0.0009). Oc. sierrensis transmission rates were significantly lower than the same six species except for Oc. dorsalis (p<0.0009).

Infection and transmission rates were lower for mosquitoes exposed to 104.9±0.1 PFU/mL of WNV (Table 3) than to the higher dose after both 7 and 14 days of incubation. After 7 and 14 days of incubation, Culex species had a wide range of infection rates, except for Cx. tarsalis (Coachella Valley) on day 14 and Cx. p. quinquefasciatus (Coachella Valley) on days 7 and 14, for which infection was not detectable. Cx. stigmatosoma infection rates on day 14 for the low dose were significantly higher than all other day-14 low–dose infection rates, except for Cx. erythrothorax (Coachella Valley) and Cx. tarsalis (Yolo County) (p<0.001). Cx. erythrothorax (Coachella Valley) infection rates on day 7 were significantly higher than all day-7 infection rates, except for Cx. tarsalis (Bakersfield), Cx. p. quinquefasciatus (Bakersfield), and Cx. p. pipiens (p<0.001). Infection rates for Cx. erythrothorax (Coachella Valley) on day 14 also were significantly higher than all day-14 low–dose infection rates except for Cx. tarsalis (Yolo County, Coachella Valley), Cx. p. pipiens, and Cx. stigmatosoma (p<0.001). Infection rates for Ochlerotatus species were <5% at 7 and 14 days of incubation.

**Table 3:** Infection and transmission rates for California mosquito species orally infected with 10^4.9±0.1^ PFU/mL of *West Nile virus* (WNV)

Species	Source by county	Day transmission attempted	No. tested	Infection rate^a^	Transmission rate^b^
*Culex tarsalis*	Yolo	7	25	8	0
		14	11	36	82
	Kern	7	10	30	10
		14	45	7	0
	Riverside	7	40	13	0
		14	10	0	0
*Cx. pipiens quinquefasciatus*	Kern	7	50	58	0
		14	50	10	0
	Riverside	7	50	0	0
		14	55	0	0
*Cx. p. pipiens*	Shasta	7	25	36	0
		14	35	23	60
*Cx. stigmatosoma*	San Bernardino	14	29	69	34
*Cx. erythrothorax*	Orange	7	47	15	0
	Riverside	7	12	67	0
		14	20	65	30
*Ochlerotatus dorsalis*	San Luis Obispo	7	29	3	0
		14	25	4	4
*Oc. melanimon*	Kern	7	50	0	0
		14	60	3	2
*Oc. sierrensis*	Lake	7	25	4	0
		14	30	0	0

^a^Percent of mosquito bodies positive for WNV. ^b^Percent of transmission attempts positive for WNV.

After imbibing a low dose of virus and undergoing 7 days of incubation, positive transmissions were not detected except for Cx. tarsalis (Bakersfield). Transmission rates were highest after 14 days of incubation for Cx. tarsalis (Yolo County), Cx. p. pipiens, Cx. stigmatosoma, and Cx. erythrothorax (Coachella Valley), although transmission rates for Cx. tarsalis (Yolo County) and Cx. p. pipiens were higher than the infection rates. Their transmission rates were significantly higher than all others (p<0.001). Transmission rates were ≤4% for Oc. dorsalis and Oc. melanimon after 14 days. WNV transmission was not detected for Cx. tarsalis (Coachella Valley), Cx. p. quinquefasciatus (Bakersfield and Coachella Valley), Cx. erythrothorax (Orange County), and Oc. sierrensis. Ae. vexans and Cs. inornata were not tested at the low dose of virus.

## Discussion

All 10 California mosquito species were competent laboratory vectors of WNV, although infection rates varied by species, dose, and incubation period. The amount of virus we used for infection was comparable to published natural WNV avian viremias in Egypt (32) but less than reported for North American birds infected with the NY strain of WNV (33). In addition, artificial blood meals with defibrinated blood may be less infectious by ~2 logs of virus/mL (34), although recent comparisons among Cx. tarsalis (infected with WEEV by feeding on viremic chickens or heparinized viremic chicken blood presented by hanging blood droplets, pledgets, or solutions through a biomembrane) did not show significant differences in infection rates or titers in infected female mosquitoes (F. Mahmood et al., unpub. data). Regardless, all mosquito species became infected and transmitted WNV at some level.

Cx. tarsalis is one of the most efficient laboratory vectors of WNV tested from North America (10,13–15). This species is abundant in California and much of western North America, where it is involved in the maintenance and amplification of WEEV and SLEV (22). Considering its central role in the transmission of arboviruses in avian hosts and its susceptibility to WNV infection in the laboratory, Cx. tarsalis has the greatest potential of the species we studied to amplify and maintain WNV in California.

Mosquitoes in the Cx. pipiens species complex also may be an important enzootic mosquito host in California. Cx. p. pipiens was identified as a primary WNV vector during the 1999 New York outbreak (10) and has been suggested as a host for overwintering flaviviruses such as WNV and SLEV (35–38). This species could play a similar role in WNV transmission in California. Cx. p. pipiens is mainly ornithophilic (39), but Cx. p. quinquefasciatus feeds readily on mammals (25,40), potentially transferring WNV from birds to humans and horses.

Cx. p. quinquefasciatus from Coachella Valley and Orange County were significantly less susceptible to infection than those collected from Bakersfield in the southern Central Valley. Differences in infection and transmission rates indicated that geographic differences may exist in the vector competence for WNV of mosquitoes within this species complex, which could relate to the introgression of Cx. p. pipiens genes into the Bakersfield population (23). The extent to which differences in infection and transmission are caused by the genetic structure of mosquito populations throughout the state and the impact of these differences on WNV transmission require additional study.

Results for Cx. tarsalis (Yolo County) and Cx. p. pipiens exposed to the low dose of virus and incubated for 14 days were unexpected. Infection rates for both species were consistent with results for most Culex species, but transmission rates were high and exceeded infection rates (i.e., some positive expectorate samples were not associated with positive results for the associated mosquito bodies, even after retesting). These incongruous results may be attributed to experimenter error. Additional replicates of these experiments may be needed to verify our results.

Infection with WNV may have increased death rates in infected female mosquitoes. In most groups, infection rates after 14 days were less than infection rates after 7 days, perhaps indicating that susceptible females died more rapidly than less susceptible or uninfected females. Most noticeable were the synchronous deaths of Cx. tarsalis in both the high- and low-dose groups from the highly susceptible Yolo County population after 10 days of incubation.

Cx. stigmatosoma and Cx. erythrothorax are widely distributed species in California and were highly susceptible to WNV infection. Cx. stigmatosoma preferentially feeds on birds and may play a role as an enzootic vector. Conversely, Cx. erythrothorax behaves as an opportunistic feeder, potentially bridging WNV transmission between birds and mammals (25).

In California, Oc. dorsalis and Oc. melanimon are involved in the transmission of WEEV among small mammals and are both laboratory-confirmed vectors of WEEV (26,27). Both species have a similar ecology and can be found in fresh water; however, Oc. dorsalis also develops in saline and alkaline habitats in coastal and southeastern California, respectively (26,41,42). Oc. melanimon plays a secondary role in the maintenance of WEEV in lagomorphs during the late summer in the Central Valley of California (43). WEEV and California encephalitis viruses have been isolated from Oc. melanimon (44,45). Oc. melanimon is an abundant pest species in the Central Valley that readily bites humans, other mammals, and (occasionally) birds (29,46). With moderate WNV transmission rates and a preference for mammalian hosts, these species have little potential to act as secondary or bridge vectors from birds to mammals.

Oc. sierrensis, a widely distributed tree hole mosquito, is a major pest in California that frequently bites humans and other mammals (28,46). However, arboviruses have not been isolated from this species to date, and its infection and transmission rates for WNV were low in the current study. Mammalian feeding preferences coupled with low vector competence for WNV indicate that this species probably would not be an enzootic or bridge vector of WNV in California.

WNV was isolated from wild Ae. vexans collected from the eastern United States during 2001 (11). Arboviruses rarely have been isolated from Ae. vexans in California (24), even though this species has been found infected with WEEV during epizootics in the central United States (47) and has been shown capable of laboratory transmission of WEEV (48) and SLEV (49) at high infectious doses. In a single trial during the current study, Ae. vexans exhibited moderate infection and transmission rates for WNV. Mammalian feeding preferences (29,45) decrease its potential as an enzootic vector for WNV in California.

Cs. inornata is a widely distributed winter mosquito in California with relatively high infection and moderate transmission rates for WNV. The species is a laboratory-confirmed vector of WEEV and SLEV viruses (49,50) and a primary horizontal and vertical vector of some bunyaviruses (51,52). We tested this species because of its potential to extend the transmission season of WNV in California beyond the November–January diapause of Cx. tarsalis (53,54). Cs. inornata primarily feeds on livestock and occasionally on birds (46,55,56) and may play a minor role in the amplification and transmission of WNV in California.

Because WNV was recently introduced into North America, little is known about the vector competence of New World mosquitoes for this invading strain of virus. Assessing the vector competence of California mosquitoes provides arbovirus surveillance and mosquito control programs with valuable information concerning the possible roles of different species in the transmission and maintenance of WNV. Our results indicated that, similar to other parts of the world, mosquitoes in the genus Culex are anticipated to be the principal enzootic mosquito hosts of WNV in California. On the basis of their vector competence and host-feeding patterns, Cx. tarsalis may be the principal vector in rural agricultural ecosystems; in addition, members of the Cx. pipiens complex and perhaps Cx. stigmatosoma will be important vectors in urban settings. If WNV becomes established in a Cx. tarsalis–passerine transmission cycle, the effect of sharing a common vector on the evolution of two closely related flaviviruses, WNV and SLEV, will be determined. The variation in WNV vector competence and other components of vectorial capacity within single mosquito species will need to be studied. Cx. erythrothorax and species in the genera Ochlerotatus and Culiseta are likely to serve as secondary or bridge vectors. Our results for Cx. p. quinquefasciatus collected in different geographic locations, however, indicate that not all mosquitoes in a single taxonomic unit will contribute equally to WNV transmission.
