# Correction, Vol. 8, No. 12

**DOI:** 10.3201/eid0903.C0903

**Published:** 2003-03

In the article, “Vector Competence of California Mosquitoes for *West Nile virus*” by Laura B. Goddard et al., errors occurred in Table 2 on page 1387. The corrected table appears below and online at http://www.cdc.gov/ncidod/eid/vol8no12/02-0536.htm

**Table 2:** Infection and transmission rates for California mosquito species orally infected with 10^7.1±0.1^ PFU/mL of West Nile virus (WNV)

Species	Source by county	Day when transmission was attempted	No. tested	Infection rate^a^	Transmission rate^b^

Culex tarsalis	Yolo	7	30	87	60
		14	1	100	100
	Kern	7	15	93	40
		14	35	74	60
	Riverside	7	49	94	10
		14	55	85	62
*Cx. pipiens quinquefasciatus*	Kern	7	50	86	4
		14	50	58	52
	Riverside	7	60	8	0
		14	60	13	2
	Orange	14	58	28	19
*Cx. p. pipiens*	Shasta	7	45	80	9
		14	50	66	36
*Cx. stigmatosoma*	San Bernardino	7	17	100	0
		14	31	100	71
*Cx. erythrothorax*	Orange	7	15	67	0
		14	48	77	19
	Riverside	7	15	100	33
		14	25	100	64
*Ochlerotatus dorsalis*	San Luis Obispo	7	30	50	13
		14	29	41	34
*Oc. melanimon*	Kern	7	50	46	18
		14	60	48	20
*Oc. sierrensis*	Lake	7	40	5	3
		14	50	14	6
*Aedes vexans*	Riverside	14	22	32	23
*Culiseta inornata*	Kern	14	28	75	21


^a^Percent of mosquito bodies positive for WNV. ^b^Percent of transmission attempts positive for WNV.

We regret any confusion these errors may have caused.

